# TiO_2_–SiO_2_ supported MnWO*_x_* catalysts by liquid-phase deposition for low-temperature NH_3_-SCR

**DOI:** 10.1098/rsos.180669

**Published:** 2019-01-30

**Authors:** Weizhe Lu, Hanfeng Lu, Zekai Zhang

**Affiliations:** Institute of Catalytic Reaction Engineering, College of Chemical Engineering, Zhejiang University of Technology, Hangzhou 310014, People's Republic of China

**Keywords:** NH_3_-SCR, MnWO_4_, TiO_2_–SiO_2_, liquid-phase deposition, low temperature

## Abstract

NH_3_-SCR is an environmentally important reaction for the abatement of NO*_x_* from different resources. MnO_2_-based catalyst has attracted significant attention due to the excellent activity. In this paper, a series of MnWO*_x_*/TiO_2_–SiO_2_ catalysts were prepared by liquid-phase deposition method. The catalysts were characterized by N_2_ adsorption/desorption, XRD, TEM, XPS, FT-IR, H_2_-TPR, TG and water adsorption capacity. The existence of SiO_2_ improved the SO_2_ and H_2_O resistance of the MnWO*_x_*/TiO_2_–SiO_2_ catalyst without decreasing the NH_3_-SCR activity. Under the reaction conditions of 260°C and 60 000 ml g_cata_ h^−1^ gas hourly space velocity (GHSV), the NO conversion was kept stable at about 95% for 140 min on stream. The excellent performance of MnWO*_x_*/TiO_2_–SiO_2_ catalyst is considered to be originated from the texture properties and active species dispersion improvement by SiO_2_ in the support and low-temperature preparation.

## 1. Introduction

Nitrogen oxides are one of the major pollutants that endanger the atmosphere air, which can cause severe environmental problems such as acid rain, smog and photochemical pollution. deNO*_x_* is a hot topic in the environmental fields in the past decades, and many researchers continue to be concerned till present. Comparing to the other routes, it is a desirable way to reduce NO*_x_* to harmless N_2_. Selective catalytic reduction of NO*_x_* via NH_3_ (NH_3_-SCR) thereby has been widely studied and applied in the elimination of nitrogen oxides [1,2].

In general, NO*_x_* are mainly released from the combustion of fossil fuels at different stationary or mobile resources. The nature of the two types of exhaust gases is quite different. The catalysts are also evolved into two series, the transition metal oxide-based catalysts for the stationary resources, and the zeolite-based catalysts for the mobile resources. V_2_O_5_-WO_3_(MoO_3_)/TiO_2_ as the representative of the vanadium-based metal oxide catalyst has been commercialized and widely used in industry [3,4]. The working temperature window of V_2_O_5_-WO_3_(MoO_3_)/TiO_2_ is mainly in the temperature range of 300–400°C, and it cannot completely remove NO*_x_* below 250°C. The working temperature window means a strict requirement for working conditions and high energy consumption [5]. Therefore, it attracts many researchers to find new catalyst systems that can operate at lower temperatures.

Among newly developed catalyst systems, Mn and Ce oxide-based catalysts have shown outstanding performance [6–11]. Especially, manganese-oxide-based catalysts can achieve very high NO*_x_* conversion even below 100°C temperature, which makes them a very competitive alternative NH_3_-SCR catalysts system [12,13]. However, the main shortcoming of Mn-based catalysts is the poor resistance to the SO_2_ in the exhaust gases. Therefore, Ce-based catalysts, especially Ce-W mixed oxides catalysts, attracted some more attention in the very recent years. At present, the commonly accepted catalyst for SO_2_ poisoning is that a large amount of sulfate is formed, which blocks the catalyst pores and inactivates the active sites. Li's and He's groups have done a lot of research and made notable progress [14–17].

Meanwhile, Mn-based catalysts are still worthy to be studied as long as the sulfur resistance is improved. By investigation, it is found that tungsten may possess the ability. Tungsten has been added and helped for the V, Ce-based catalysts, and even for the NiWS catalyst in the hydrodesulfurization process [18,19]. Liu *et al.* first considered MnWO*_x_* as the main active phase and got high deNO*_x_* efficiency from 60 to 250°C [20]. Sun *et al.* then prepared a series of W*_α_*Mn_1−*α*_O*_x_* catalysts via coprecipitation method [21]. W_0.33_Mn_0.66_O*_x_* catalyst with amorphous or poorly crystalline Mn and W species showed the highest NH_3_-SCR activity within a broad temperature range of 230–470°C. Our group also prepared the MnWTiO_2−δ_ catalyst with MnWO_4_ crystal structure and obtained a high activity in the range of 200–400°C [22,23].

The deactivation of the NH_3_-SCR catalyst mainly comes from two aspects, the poisoning caused by the reaction of SO_2_ with the active components, and the coverage or blocking (coking) of the surface of the ammonium sulfate [24–26]. Owing to the viscosity of NH_4_HSO_4_ and (NH_4_)_2_SO_4_, it is easy to bind to the catalyst and reduce the specific surface area. The formation of NH_4_HSO_4_ and (NH_4_)_2_SO_4_ often needs water. If the surface has well-hydrophobic properties, it would be possible to reduce the water adsorption and thus decrease the formation of NH_4_HSO_4_ and (NH_4_)_2_SO_4_; i.e. the sulfur resistance of the catalyst is expected to be improved by increasing the hydrophobicity of the catalyst surface [27,28].

In this work, a series of TiO_2_–SiO_2_-supported MnMO*_x_* catalysts were prepared by a liquid-phase deposition (LPD) method. The introduction of SiO_2_ increases the specific surface area of the catalyst; moreover, probably by the reaction of manganese nitrate and ammonia tungstate to form MnWO_4_, the catalysts could be prepared at relatively low temperature, which greatly improves the activity and N_2_ selectivity of the MnWO*_x_*/TiO_2_–SiO_2_ catalyst as well as the SO_2_ and H_2_O resistance.

## 2. Experimental procedure

### 2.1. Catalyst preparation

MnWO*_x_*/TiO_2_–SiO_2_ catalysts were prepared by two main procedures. The first step is to prepare TiO_2_–SiO_2_ support by a sol–gel method. The method used TEOS (*n*-ethyl silicate) and TBOT (tetrabutyl titanate) as precursors. Taking 10% SiO_2_–90% TiO_2_ as an example, a suitable amount of TEOS (1.0 ml) was mixed in anhydrous ethanol, then a small amount of distilled water (about 2.0 ml) and several drops of 1 mol l^−1^ HCl were added stirring for 30 min at room temperature to adjust pH = 2 and got solution A. Meanwhile, 5.0 ml of acetic acid was mixed with 49.0 ml anhydrous ethanol, then a small amount of distilled water (about 2.0 ml) and several drops of 1 mol l^−1^ HCl were added to adjust pH ≤ 3 and got solution B. Solutions A and B were then uniformly mixed by stirring to get solution C. Then 13.5 ml anhydrous ethanol and 13.5 ml TBOT were uniformly mixed to get solution D. Solution D was added into solution C at a rate of 3 ml min^−1^ under vigorous stirring at room temperature. The resulting material was dried in an oven at 80°C for 12 h to obtain the TiO_2_–SiO_2_ dry gel. The gel was ground into powder to obtain the support.

Considering the dispersion of active components on the hydrophobic support, a modified liquid deposition method was used to load the MnWO*_x_* on the TiO_2_–SiO_2_. Manganese nitrate solution (50% Mn(NO_3_)_2_) and tungaline ((NH_4_)_10_W_12_O_41_ · *x*H_2_O) were used as precursors; 0.845 g (NH_4_)_10_W_12_O41 · *x*H_2_O and 2.386 g Mn(NO_3_)_2_ solution were dissolved with the same molar oxalic acid in deionized water. TiO_2_–SiO_2_ powder was added to the solution and the mixture was vigorous stirred for 1 h. Ammonia water (0.5 mol l^−1^) was slowly added into the mixture to adjust the pH to 10 to achieve the precipitation of active species. The mixture was filtered, washed and the precipitate in the oven was dried at 110°C for 12 h. At last, the sample was moved into the muffle and calcined at 200°C for 2 h to obtain the target MnWO*_x_*/TiO_2_–SiO_2_ catalyst. The samples were labelled as Mn*_x_*W*_y_*O*_x_*/TiO_2_–SiO_2_(*n*), where *x* and *y* are the molar ratio of Mn : W and *n* is the percentage of SiO_2_ to (SiO_2_ + TiO_2_).

### 2.2. Catalyst characterization

N_2_ adsorption/desorption of the catalysts were measured by a Micromeritics 3Flex physical adsorption instrument. The samples were heated to 200°C under vacuum pressure and kept for 10 h before measurement. Specific surface area was calculated using the BET method.

Crystal structure of the catalysts were detected by an ARL SCINTAG X'TRA instrument (Shimadzu.) using Cu K*α* radiation in 2*θ* range of 10–80° with a step size of 0.02°.

H_2_ temperature-programmed reduction (H_2_-TPR) was conducted on a chemical adsorption apparatus (Finetec Corp.). The samples were pretreated at 400°C in Ar for 40 min. The TPR analysis was carried out in a reducing gas mixture (30 ml min^−1^) consisting of 5% H_2_ and balance Ar from 60 to 800°C at a rate of 10°C min^−1^. TCD detector temperature was 60°C.

X-ray photoelectron spectroscopy (XPS) was conducted on a Kratos AXIS Ultra DLD clutches spectrophotometer. Excitation source was the monochromatic Al K*α* radiation (h*υ* = 1486.6 eV). The power was 45 W. The working voltage was 15 kV. Scanning area was 300 × 700 µm. Vacuum test was better than the 8.5 × 10^−9^ mbar. The data were corrected using the C1 s 284.8 eV as the standard.

Skeleton FT-IR spectra of the catalysts were recorded using BRUKER VERTEX70 FT-IR apparatus, with the sample in KBr pellets.

Morphologies of the catalysts were disclosed by a Philips-FEI company Tecnai G2F30 S-Twin type high-resolution transmission electron microscopy. The samples were solved in ethanol solution to get the dispersed particles. The accelerating voltage was 300 kV.

The water adsorption capacity of the catalysts was determined by the saturated water adsorption experiment. The appropriate amount of dry sample was put into a weighing bottle and placed in a saturated water vapour environment. After the adsorption is balanced, the mass change was weighed with a electronic balance.

The coke of the catalyst after the reaction was analysed by an STA449 integrated thermal analyser. A total of 0.100 g of the sample was placed in an alumina crucible and heated from room temperature to 1000°C at a rate of 20°C min^−1^ in a 20% O_2_/80% N_2_ atmosphere.

### 2.3. Catalytic activity tests

NH_3_-SCR activity was tested in a fixed-bed quartz tube reactor (i.d. = 8 mm). The reaction conditions were as follows: 500 ppm of NO, 500 ppm of NH_3_, 5.0% O_2_ and N_2_ as balance, 10 vol% H_2_O (when used), 100 ppm SO_2_ (when used). Total gas flow rate was about 500 ml min^−1^, and calculated gas hourly space velocity (GHSV) of 60 000 ml g_cata_ h^−1^. Outlet gases were measured by an online MODEL T200H/M nitrogen analyser and Thermo trace 1300 gas chromatography. NO conversion (*X*) and N_2_ selectivity (*S*) were calculated by the following equations:

2.1 $$X(\text{\%})=\frac{\left({\left[\mathrm{NO}\right]}_{\mathrm{in}}-{\left[\mathrm{NO}\right]}_{\mathrm{out}}\right)}{{\left[\mathrm{NO}\right]}_{\mathrm{in}}}$$

and

2.2 $$S(\%)=\frac{\left(({\left[\text{NO}\right]}_{\text{in}}+{\left[{\text{NH}}_{3}\right]}_{\text{in}})-{\left[{\text{NO}}_{2}\right]}_{\text{out}}-2{\left[{\text{N}}_{2}\text{O}\right]}_{\text{out}}\right)}{\left({\left[\text{NO}\right]}_{\text{in}}+{\left[{\text{NH}}_{3}\right]}_{\text{in}}\right)}.$$

## 3. Results and discussions

### 3.1. NH_3_-SCR activity of MnWO*_x_*/TiO_2_–SiO_2_ and effect of preparation conditions

Prior to this paper, the MnWO*_x_*/TiO_2_ catalysts are mainly prepared by self-propagating high-temperature synthesis method (SHS) in our group, because the as-prepared samples possess very good properties such as narrow particle distribution and uniformly distributed MnWO*_x_* species on the TiO_2_ support. However, it seems that the method is not very suitable for TiO_2_–SiO_2_ support. The sample temperature could rise to very high (greater than 1000°C) during the preparation process and affect the pore structure of the TiO_2_–SiO_2_ support.

**Figure 1.**
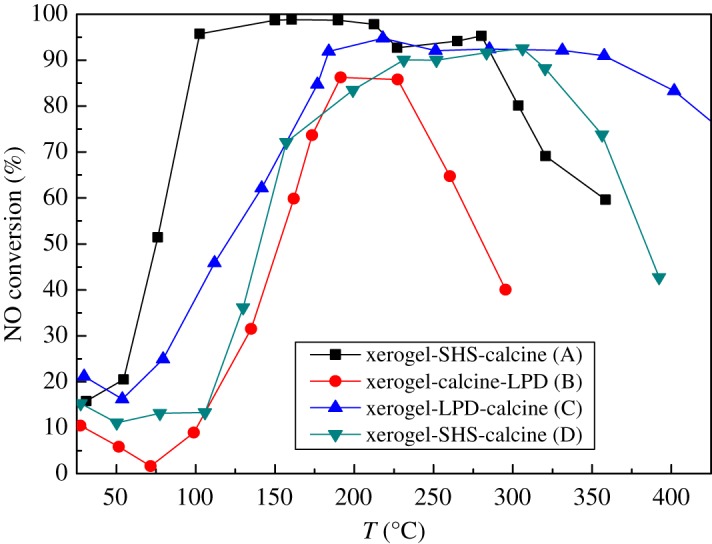
NH_3_-SCR activity of Mn_2_WO*_x_*/TiO_2_–SiO_2_ with different preparation steps.

Figure 1 shows that the activity of Mn_2_WO*_x_*/TiO_2_–SiO_2_(10) is somewhat worse than that of the Mn_2_WO*_x_*/TiO_2_ with the same SHS procedure. Therefore, a modified liquid deposition method is used to prepare the Mn_2_WO*_x_*/TiO_2_–SiO_2_ catalysts later. LPD is a method that through the reaction of the precursors salts solution forms a precipitate and deposit on the solid support pre-placed in the solution to realize the uniform loading of active components. It has been found that Mn(NO_3_)_2_ and (NH_4_)_10_W_12_O_41_ can not only react to produce a precipitation in the solution, but also form a MnWO_4_ structure in the precipitation [29], which is considered to be favourable for the activity of Mn_2_WO*_x_*/TiO_2_ catalysts. Here, the method is also adopted to prepare Mn_2_WO*_x_*/TiO_2_–SiO_2_. The sample exhibits rather high activity in NH_3_-SCR reaction. The NO conversion reaches 90% from 200°C to about 400°C, which is even broader than the results of the Mn_2_WO*_x_*/TiO_2_ by SHS method.

The preparation procedure has a clear influence on the activity of Mn_2_WO*_x_*/TiO_2_–SiO_2_ by LPD method. If the TiO_2_–SiO_2_ gel was calcined at 500°C before the LPD, the activity of the as-prepared catalyst would decrease. That is to say, the NO conversion only keeps above 90% in a narrow range of 200–230°C. It is because, after calcination, the pore structure of TiO_2_–SiO_2_ gel is decreased to some extent and the hydrophobicity increases, which is not favourable for the dispersion and deposition of active components. Nonetheless, the LPD method can effectively load MnWO_*x*_ active species on the TiO_2_–SiO_2_ support and keep the activity of Mn_2_WO*_x_*/TiO_2_–SiO_2_ catalyst in the right preparation procedure and conditions.

**Figure 2.**
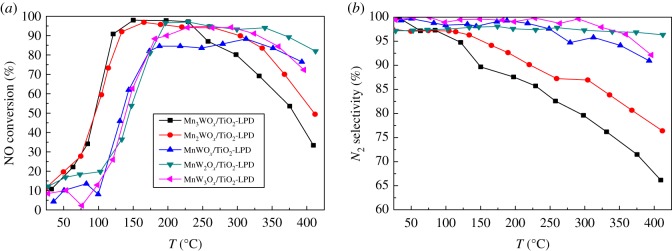
NH_3_-SCR activity of MnWO*_x_*/TiO_2_–SiO_2_ with different Mn : W ratios.

Figure 2*a* shows the NH_3_-SCR activity and figure 2*b* shows the N_2_ selectivity of the Mn*_x_*W_y_O*_x_*/TiO_2_–SiO_2_ catalysts with different Mn : W ratios synthesized by LPD. It can be seen that the Mn : W ratio influences the temperature window of the MnWO*_x_*/TiO_2_–SiO_2_ catalysts. Taking 90% NO conversion as the criterion, the working temperature window of the Mn*_x_*W*_y_*O*_x_*/TiO_2_–SiO_2_ catalysts varies from 120 to 200°C, 120 to 300°C, 140 to 280°C, 200 to 340°C and 200 to 370°C when the Mn : W ratio decreases from 3 : 1, 2 : 1, 1 : 1, 1 : 2 and 1 : 3, respectively. Taking Mn : W ratio of 1 : 1 as a dividing line, when the manganese content is rich, the catalyst has better activity at low temperatures and worse activity at high temperatures; while when the tungsten is rich, it is just in the opposite way. In general, the Mn_2_WO*_x_*/TiO_2_–SiO_2_ shows the widest working temperature window.

Comparing to activity, Mn : W ratio has a more pronounced effect on N_2_ selectivity of the catalyst. In figure 2*b*, over the MnW_2_O*_x_*/TiO_2_–SiO_2_ and MnW_3_O*_x_*/TiO_2_–SiO_2_, the N_2_ selectivity is almost unchanged with the temperature rising; while over the other samples, the N_2_ selectivity is decreased from about 100% to 87%, 82% and 75% when the Mn : W ratio increases from 1 : 1 to 3 : 1, respectively. The results indicate that Mn element is favourable for the low-temperature activity and W element is favourable for the N_2_ selectivity. It is desirable to maintain a certain manganese tungsten ratio for the MnWO*_x_*/TiO_2_–SiO_2_ catalyst to get a satisfying activity and N_2_ selectivity.

**Figure 3.**
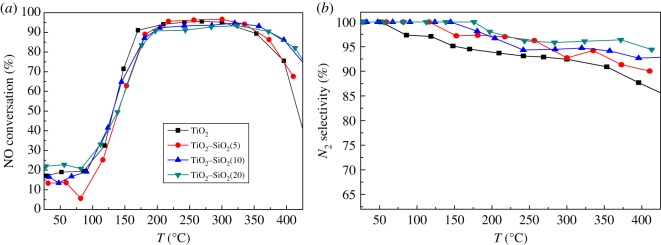
NH_3_-SCR activity of MnWO*_x_*/TiO_2_–SiO_2_ with different TiO_2_ : SiO_2_ per cents.

The support properties often can be adjusted by the composite variation and influence the catalyst properties. TiO_2_–SiO_2_ oxides with different SiO_2_ contents from 5% SiO_2_/95% TiO_2_ to 20% SiO_2_/80% TiO_2_, which are labelled as TiO_2_–SiO_2_(5), TiO_2_–SiO_2_(10) and TiO_2_–SiO_2_(20), are studied. Figure 3*a* shows the results of the activities of the samples in the NH_3_-SCR reaction. Taking 90% NO conversion as criterion, the increase of SiO_2_ content in the support does not change the working temperature window of Mn_2_WO*_x_*/TiO_2_–SiO_2_ too much. All the low-temperature boundaries of the working temperature windows are around 170°C. When the SiO_2_ content increases from 5% to 20%, it only makes the high-temperature boundary move a slight degree to the high-temperature direction, and slightly widens the working temperature window. The working temperature windows of Mn_2_WO*_x_*/TiO_2_–SiO_2_(10) and Mn_2_WO*_x_*/TiO_2_–SiO_2_(20) are the same as 170–370°C.

The addition of SiO_2_ also has a positive effect on the N_2_ selectivity of the catalyst, as shown in figure 3*b*. It can be found that N_2_ selectivity on the Mn_2_WO*_x_*/TiO_2_ (without SiO_2_) catalyst at 50°C began to decrease rapidly with the increase of temperature. Nonetheless, the N_2_ selectivity can be maintained at above 90% on all the Mn_2_WO*_x_*/TiO_2_–SiO_2_ samples. Further, as the SiO_2_ content increases, N_2_ selectivity decreases slowly, and it decreases the slowest on the Mn_2_WO*_x_*/TiO_2_–SiO_2_(20) sample.

### 3.2. The resistance of MnWO*_x_*/TiO_2_–SiO_2_ to H_2_O and SO_2_

**Figure 4.**
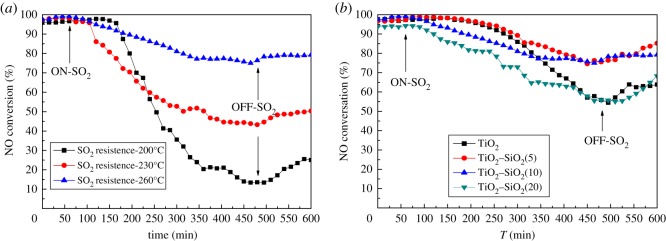
NH_3_-SCR activity of Mn_2_WO*_x_*/TiO_2_–SiO_2_ in 100 ppm SO_2_.

As is known, the flue gas usually contains SO_2_ and water vapour, which often affects the NH_3_-SCR catalyst performance. It is the primary reason that the Mn_2_WO*_x_*/TiO_2_ catalyst is modified by SiO_2_ in this paper. Figure 4 shows the NH_3_-SCR results of Mn_2_WO*_x_*/TiO_2_–SiO_2_ catalysts with different SiO_2_ content in 100 ppm SO_2_ at different reaction temperature. The SO_2_ is introduced after 60 min of reaction time on stream.

It can be seen that SiO_2_ has a significant effect on the sulfur resistance of the Mn_2_WO*_x_*/TiO_2_–SiO_2_ catalyst. Taking the introduction time of SO_2_ flow (60 min) as the initial point, for the Mn_2_WO*_x_*/TiO_2_ catalyst, the NO conversion begins to decrease after 90 min (total 150 min time on stream) reaction after the introduction of SO_2_ flow. It decreases with a slow rate till 240 min (total 300 min), and then decreases with a significant rate to 55% at 420 min (total 480 min), where SO_2_ is switched off. The NO conversion then rises back to about 65% in SO_2_-free flow after 120 min (total 600 min on stream). The performance of Mn_2_WO*_x_*/TiO_2_–SiO_2_(5) was similar to that of Mn_2_WO*_x_*/TiO_2_ catalyst before 240 min, but the NO conversion decreased significantly slower than the Mn_2_WO*_x_*/TiO_2_ catalyst. The NO conversion is still close to 80% at 420 min, and after SO_2_ switches off for 120 min, it rises back to close to 90% again. For the catalyst Mn_2_WO*_x_*/TiO_2_–SiO_2_(10), the NO conversion decreases with a rather slow rate after 30 min of SO_2_ flow introduction in a linear style till 300 min, where the NO conversion is kept at about 80%. In the remaining time, no significant change is observed even though the SO_2_ is switched off. Finally, for the catalyst Mn_2_WO*_x_*/TiO_2_–SiO_2_(20), the NO conversion decreases clearly in a different way from other samples. A number of stable platforms appeared on the profile, mainly in the range of 160–220 min, 260–300 min and 330–420 min total time on stream. When the SO_2_ is switched off, the NO conversion can rise back to about 70% after 120 min. The results show that the appropriate amount of SiO_2_ is beneficial to improve the sulfur resistance of the Mn_2_WO*_x_*/TiO_2_–SiO_2_ catalyst.

To further evaluate the effect, the performance of Mn_2_WO*_x_*/TiO_2_–SiO_2_(10) is then tested under 230°C and 200°C. However, the results in figure 4*b* are not very good. At 230°C, NO conversion has dropped below 50% after 420 min on stream; and at 200°C, NO conversion only is left no more than 20%. It means that the effect of SiO_2_ is still limited and the catalyst needs to be more improved in the future.

**Figure 5.**
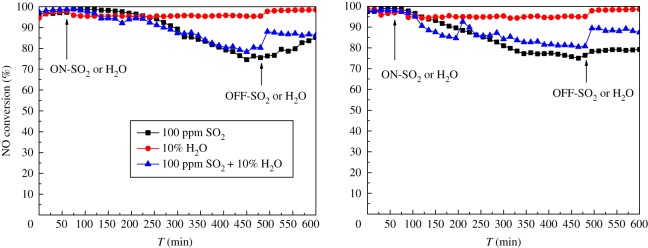
NH_3_-SCR activity of Mn_2_WO*_x_*/TiO_2_–SiO_2_(5) and Mn_2_WO*_x_*/TiO_2_–SiO_2_(10) at 260°C in the presence of H_2_O, SO_2_ and SO_2_ + H_2_O.

Next, the activity of Mn_2_WO*_x_*/TiO_2_–SiO_2_(5) and Mn_2_WO*_x_*/TiO_2_–SiO_2_ (10) at 260°C in the presence of 10% H_2_O and SO_2_ + H_2_O together are investigated in figure 5. Both of the NO conversions can maintain above 95% in the presence of 10% H_2_O. After the H_2_O is switched off, the activity of the catalyst can be fully restored in a short time, indicating that the catalyst has good resistance to H_2_O. In the 10 vol% H_2_O and 100 ppm SO_2_ atmosphere, the NO conversion profile on the Mn_2_WO*_x_*/TiO_2_–SiO_2_(5) is similar with that in the 100 ppm SO_2_ flow, while its decrease degree is a little slight. The NO conversion is kept above 80% at 480 min. When H_2_O and SO_2_ are stopped, NO conversion rate rises rapidly. As for the Mn_2_WO*_x_*/TiO_2_–SiO_2_(10), the NO conversion quickly decreases to about 80% and keeps at that level till 480 min. When the H_2_O and SO_2_ is switched off, it also rises back to about 90% in a short time. That is to say, both of the samples show good resistance to the H_2_O and SO_2_.

### 3.3. Catalysts characterization

Table 1 lists some texture properties such as the specific surface area, the average pore size and pore volume of the TiO_2_–SiO_2_ supports and the Mn_2_WO*_x_*/TiO_2_–SiO_2_ catalysts prepared by sol–gel method and LPD. It can be found that the addition of SiO_2_ clearly increases the specific surface area of TiO_2_–SiO_2_ gels comparing to TiO_2_. With the increase of SiO_2_ amount, the specific surface area of TiO_2_–SiO_2_(5), TiO_2_–SiO_2_(10) and TiO_2_–SiO_2_(20) increases from 263.9 and 283.5 to 332.6 m^2^ g^−1^; the pore volume increases from 0.145 and 0.158 to 0.213 cm^3^ g^−1^, respectively. While the average pore size is kept at about 2.2 nm, only TiO_2_–SiO_2_(20) is increased to 2.6 nm. When the active species are loaded on the TiO_2_–SiO_2_ support, all the specific surface areas and pore volumes of Mn_2_WO*_x_*/TiO_2_–SiO_2_ catalysts are decreased, while the average pore sizes are increased. The Mn_2_WO*_x_*/TiO_2_–SiO_2_(5) catalyst can be found to have the best pore structure and keep the largest specific surface area of 235.9 m^2^ g^−1^, the average pore diameter of 2.9 nm and pore volume of 0.170 cm^3^ g^−1^.

**Table 1.** The texture properties of different catalysts and supports before reaction.

samples	BET surface area (m^2^ g^−1^)	average pore diameter (nm)	pore volume ( cm^3^ g^−1^)
Mn_2_WO*_x_*/TiO_2_–SiO_2_(20)	219.5	2.7	0.150
Mn_2_WO*_x_*/TiO_2_–SiO_2_(10)	219.6	2.6	0.142
Mn_2_WO*_x_*/TiO_2_–SiO_2_(5)	235.9	2.9	0.170
Mn_2_WO*_x_*/TiO_2_	184.1	2.9	0.135
TiO_2_–SiO_2_(20)	332.6	2.6	0.213
TiO_2_–SiO_2_(10)	283.5	2.2	0.158
TiO_2_–SiO_2_(5)	263.9	2.2	0.145
TiO_2_	215.7	2.2	0.120

The texture properties of the heterogenous catalyst are often changed after the reaction. To investigate the properties of Mn_2_WO*_x_*/TiO_2_–SiO_2_ catalysts before and after the NH_3_-SCR reaction in the different atmospheres, table 2 lists their texture properties after the NH_3_-SCR reaction with/without H_2_O and SO_2_ flow.

**Table 2.** The texture properties of different catalysts after reactions.

samples	BET surface area (m^2^ g^−1^)	average pore diameter (nm)	pore volume (cm^3^ g^−1^)
Mn_2_WO*_x_*/TiO_2_–SiO_2_(20)–SO_2_	147.7	2.8	0.104
Mn_2_WO*_x_*/TiO_2_–SiO_2_(20)–H_2_O	208.7	2.7	0.143
Mn_2_WO*_x_*/TiO_2_–SiO_2_(20)–SO_2_ + H_2_O	140.5	2.8	0.099
Mn_2_WO*_x_*/TiO_2_–SiO_2_(10)–SO_2_	177.2	2.7	0.120
Mn_2_WO*_x_*/TiO_2_–SiO_2_(10)–H_2_O	210.2	2.6	0.136
Mn_2_WO*_x_*/TiO_2_–SiO_2_(10)–SO_2_ + H_2_O	167.8	2.6	0.109
Mn_2_WO*_x_*/TiO_2_–SiO_2_(5)–SO_2_	187.9	2.7	0.129
Mn_2_WO*_x_*/TiO_2_–SiO_2_(5)–H_2_O	221.4	2.9	0.161
Mn_2_WO*_x_*/TiO_2_–SiO_2_(5)–SO_2_ + H_2_O	168.6	2.8	0.118
Mn_2_WO*_x_*/TiO_2_–SO_2_	125.2	3.1	0.096
Mn_2_WO*_x_*/TiO_2_–H_2_O	174.3	2.9	0.124
Mn_2_WO*_x_*/TiO_2_–SO_2_ + H_2_O	116.5	3.1	0.087

The presence of water has little effect on the texture properties of the catalysts. All the average pore sizes do not change after reaction, and the specific surface area of the catalysts decreases about 10 m^2^ g^−1^. SO_2_ significantly affects the nature of the catalysts. All the specific surface area and pore volumes of the catalysts after the reaction are largely reduced. The largest decrement is from Mn_2_WO*_x_*/TiO_2_–SiO_2_(20). The specific surface area decreases 71.8 m^2^ g^−1^, and the pore volume decreases 0.046 cm^3^ g^−1^. The second is the Mn_2_WO*_x_*/TiO_2_, 67.3 m^2^ g^−1^ and 0.048 cm^3^ g^−1^. Mn_2_WO*_x_*/TiO_2_–SiO_2_(10) and Mn_2_WO*_x_*/TiO_2_–SiO_2_(5) show somewhat better resistance to SO_2_. Their specific surface areas only decrease 42.4 and 48 m^2^ g^−1^. Especially, the pore volume of Mn_2_WO*_x_*/TiO_2_–SiO_2_(10) only decreases 0.022 cm^3^ g^−1^. The average pore size of Mn_2_WO*_x_*/TiO_2_–SiO_2_(5) sample is the only one enlarged after the reaction. Coexistence of water and SO_2_ does not show a synergistic effect on the nature of the catalysts. The change in the values is close to the sum of the influence of the two compounds.

**Figure 6.**
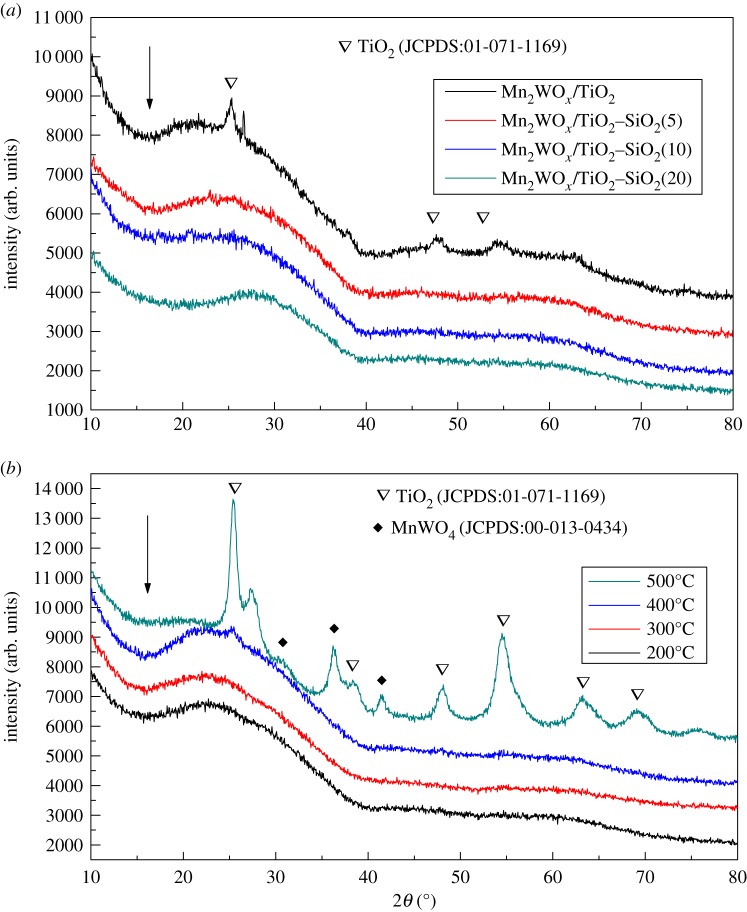
XRD patterns of Mn_2_WO*_x_*/TiO_2_–SiO_2_ catalysts.

Figure 6 shows the XRD pattern of the Mn_2_WO*_x_*/TiO_2_–SiO_2_ catalysts. It can be seen that for the as-prepared Mn_2_WO*_x_*/TiO_2_–SiO_2_ catalysts, except the profile of Mn_2_WO*_x_*/TiO_2_ sample shows the characteristics peaks of 2*θ* = 25.2°, 48.1°, 54.0° and 62.8° attributed to the anatase TiO_2_, neither the characteristic peaks of active species such as MnO*_x_*, WO_3_ and MnWO*_x_*, nor the characteristic peaks of support such as the anatase TiO_2_ and rutile TiO_2_ or SiO_2_ appear on the other samples. The results may indicate the uniformity of the crystal sizes of the support and active species carrier are uniform and small, but it may also mean that the resulting catalysts are predominantly the amorphous species.

**Figure 7.**
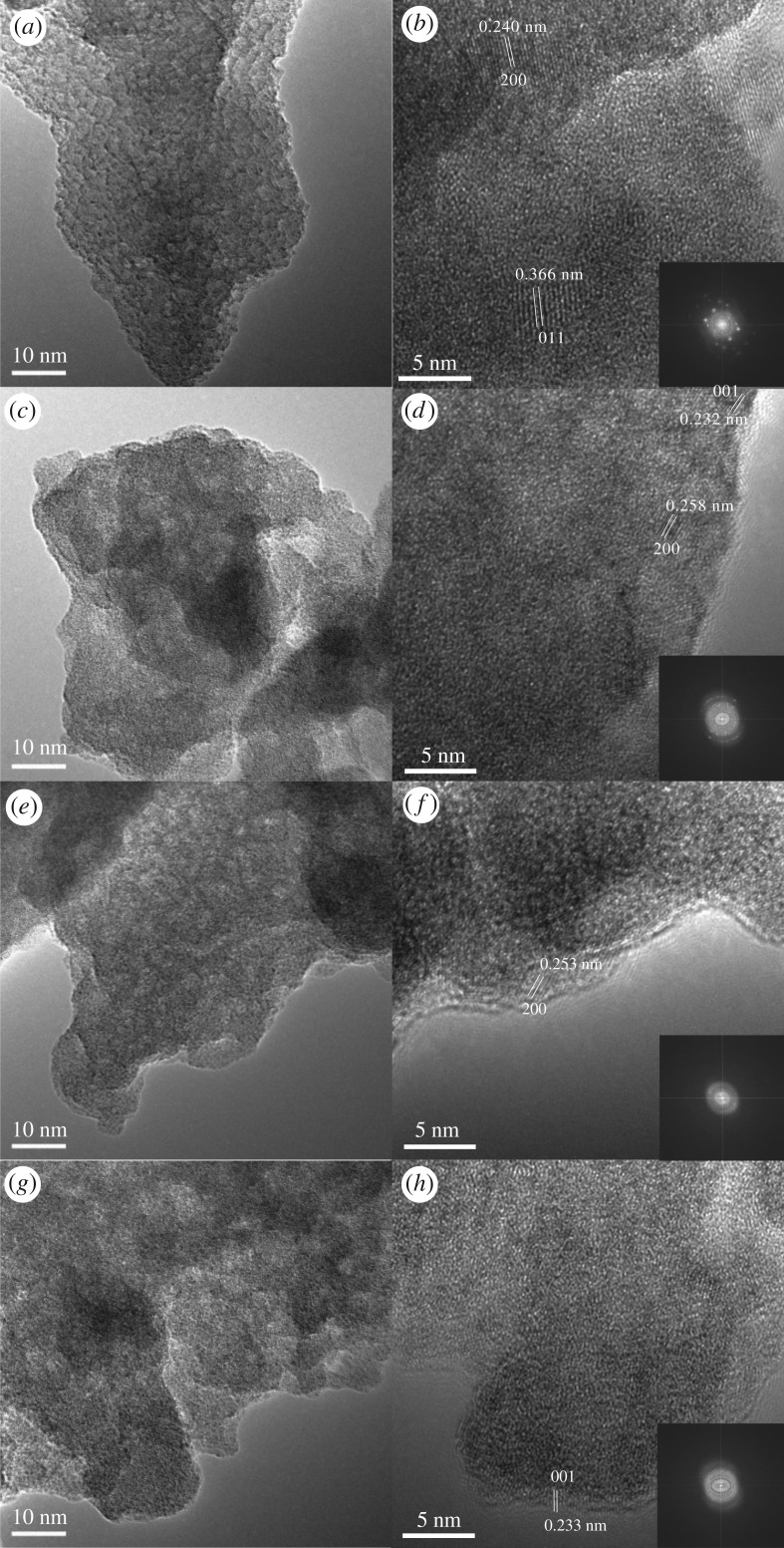
TEM images of the Mn_2_WO*_x_*/TiO_2_–SiO_2_ catalysts. (*a*,*b*) Mn_2_WO_*x*_/TiO_2_, (*c*,*d*) Mn_2_WO_*x*_/TiO_2_–SiO_2_(5), (*e,f*) Mn_2_WO_*x*_/TiO_2_–SiO_2_(10), (*g*,*h*) Mn_2_WO_*x*_/TiO_2_ -SiO_2_(20).

Figure 7 then exhibits the morphologies of the catalysts by TEM. From the images, on the surface of the silicon-free Mn_2_WO*_x_*/TiO_2_, there are clear crystal lattice fringes, which can be identified and attributed to the different crystal faces of the active species or the supports. The 0.345 nm lattice spacing is from (101) face of TiO_2_; 0.240 nm and 0.366 nm spacing can be attributed to with (200) and (011) face of MnWO_4_ crystal. It is similar to our previous results obtained by SHS method [22]. The results confirmed that the reaction to form manganese tungstate has occurred in the solution. The sample has well crystallinity with a uniform particles size distribution. Meanwhile, for the silicon-containing samples, the particles are also relatively small. But the lattice fringes are not very clear due to the high contents of the mixed crystal composition, which means that the SiO_2_ affects the crystallinity of the MnWO*_x_* active species.

**Figure 8.**
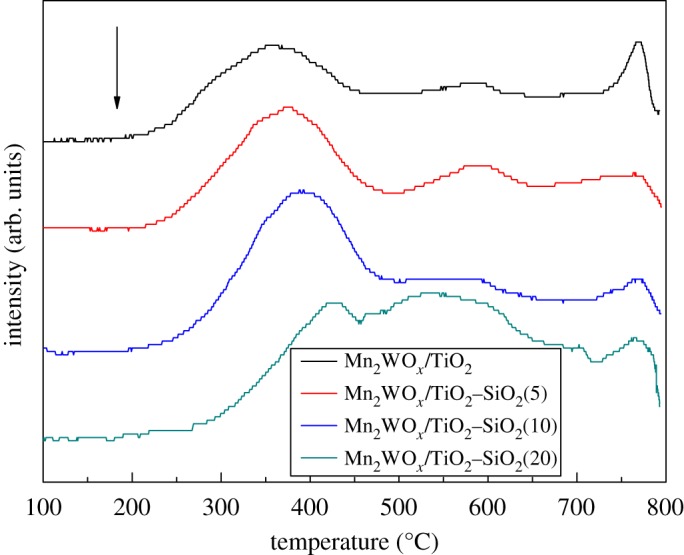
H_2_-TPR profiles of the Mn_2_WO*_x_*/TiO_2_–SiO_2_ catalysts.

Figure 8 shows the H_2_-TPR profiles of Mn_2_WO*_x_*/TiO_2_–SiO_2_ catalysts with different SiO_2_ contents. The reduction peaks may originate from the different active species and supports, which leads to the profiles somewhat complicated. In general, the profile can be divided into two reduction bands and one big peak. According to the previous discussions [30–32], for the Mn_2_WO*_x_*/TiO_2_ sample, in the lower temperature range of 250–480°C, the reduction bands or peaks are mainly originated from different MnO*_x_* species; in the higher temperature range of 500–800°C, the reduction band and peak are from different Ti or W oxides species. More detailed, the identified reduction peaks at about 300°C and 340°C are attributed to the reduction peaks of MnO_2_ → Mn_3_O_4_ and Mn_3_O_4_ → MnO. The reduction band of 520°C–600°C is the contribution of Ti^4+^ → Ti^3+^, and the peak at 780°C is from W^6+^ → W^4+^. When the SiO_2_ is introduced into TiO_2_, there are several significant changes observed. The first is that the reduction band in the low-temperature range moves to the high-temperature attitude; the second is the strength of the reduction band in the middle temperature increases and the peak at 780°C decreases. The movement of the band in the low-temperature range means that the MnO*_x_* species are inclined to be hard to reduce, which shall not be favourable for the NH_3_-SCR activity of the Mn_2_WO*_x_*/TiO_2_–SiO_2_ catalysts. However, the NH_3_-SCR activity seems to be not influenced clearly. It might be amended by the dispersion increment of active species by the TiO_2_–SiO_2_ support. The strength increment of the peak at the middle temperature illustrates that more TiO_2_ can be reduced. The TiO_2_ crystal structure might be broken and more amorphous or isolated species have been formed.

**Figure 9.**
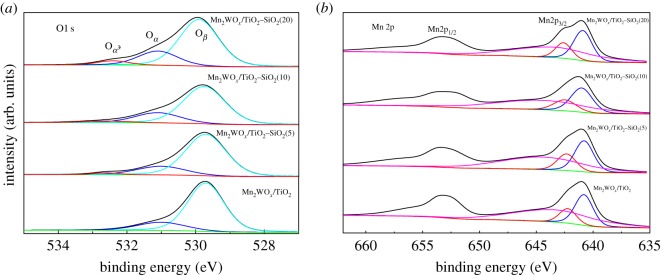
O1 s (*a*) and Mn 2p (*b*) XPS spectra of the Mn_2_WO*_x_*/TiO_2_–SiO_2_ catalysts.

In order to investigate the valence state of the Mn species and O species, XPS characterization was performed for the samples. Figure 9*a* shows the O1 s spectra of Mn_2_WO*_x_*/TiO_2_, Mn_2_WO*_x_*/TiO_2_–SiO_2_(5), Mn_2_WO*_x_*/TiO_2_–SiO_2_(10) and Mn_2_WO*_x_*/TiO_2_–SiO_2_(20), and table 3 lists the quantitative results via Gauss deconvolution method. After deconvolution, three sub-bands can be found on the spectra. As previous reports, the sub-bands around 529.7 eV can be attributed to the lattice oxygen (denoted as O*_β_*); the sub-bands around 531.1 eV to surface absorbed oxygen (denoted as O*_α_*) such as O_2_^2−^ and O^−^, and the sub-band around 533.2 eV can be assigned to chemisorbed water (denoted as O*_α′_*) [17,33]. With the SiO_2_ introduction increases, the peak of O*_β_* decreases and the peak of O*_α_* and O*_α′_* increases gradually. As the O*_α_* is usually regarded as more reactive in redox reactions due to its higher mobility than lattice oxygen, the percentages of O*_α_*, O*_β_* and O*_α′_* are often viewed as an indicator for the redox ability of the catalyst. From table 3, the O*_α_* per cent increases gradually from 18.32% of the Mn_2_WO*_x_*/TiO_2_ to 22.20% of Mn_2_WO*_x_*/TiO_2_–SiO_2_(20). Combining the results of XRD and TEM, it may infer that the addition of SiO_2_ decreases the crystallinity of the active species, increases the dispersion, and forms more surface active sites. Some reports mentioned that the sub-band around 529.7 eV could be attributed to Ti–O–Ti bonds; the sub-band around 532.1 eV to Si–O–Ti cross-linking bonds, and the sub-band around 533.2 eV to Si–O–Si bonds [34,35]. When the SiO_2_ introduction increases, the peak of Ti–O–Ti bonds decreases and the peak of Si–O–Ti cross-linking bonds and Si–O–Si bonds increases gradually.

**Table 3.** Quantitative results of O1 s XPS spectra of Mn_2_WO*_x_*/TiO_2_–SiO_2_ (0–20%).

	O*_β_*		O*_α_*		O*_α_*_′_	
samples	BE (eV)	Per. (%)	BE (eV)	Per. (%)	BE (eV)	Per. (%)
Mn_2_WO*_x_*/TiO_2_	529.7	81.67	531.0	18.32	532.6	0
Mn_2_WO*_x_*/TiO_2_–SiO_2_(5)	529.7	78.40	531.0	19.47	532.6	2.13
Mn_2_WO*_x_*/TiO_2_–SiO_2_(10)	529.7	74.82	531.1	21.84	532.6	3.34
Mn_2_WO*_x_*/TiO_2_–SiO_2_(20)	529.9	72.60	531.1	22.20	532.4	5.21

Figure 9*b* then shows the Mn 2p XPS spectra of the four samples and table 4 lists the quantitative results. Similarly, three sub-bands belonging to Mn^3+^ at 640.6–641.4 eV, Mn^4+^ at 641.9–642.3 eV and Mn^2+^ at 643.4–644.5 eV can be found on the spectra. According to the quantitative analysis, the Mn species with different valence states does not change clearly, and the average valence state does not change, which may indicate that the SiO_2_ only improves the dispersion of the active species, and has not much interaction effect with the active species after loading.

**Table 4.** Quantitative results of Mn2p XPS spectra of Mn_2_WO*_x_*/TiO_2_–SiO_2_ (0–20%).

	Mn^4^^+^		Mn^3+^		Mn^2+^		
samples	BE (eV)	Per. (%)	BE (eV)	Per. (%)	BE (eV)	Per. (%)	Average valence
Mn_2_WO*_x_*/TiO_2_	642.2	13.33	640.8	30.55	643.4	56.11	2.6
Mn_2_WO*_x_*/TiO_2_–SiO_2_(5)	642.3	15.86	640.8	30.24	644.5	53.89	2.6
Mn_2_WO*_x_*/TiO_2_–SiO_2_(10)	642.5	14.68	641.0	31.42	644.5	53.89	2.6
Mn_2_WO*_x_*/TiO_2_–SiO_2_(20)	642.6	15.08	640.9	32.89	643.5	51.93	2.6

At the same time, O*_α_*^,^ increment means that the introduction of SiO_2_ increases the adsorption of water on the Mn_2_WO*_x_*/TiO_2_–SiO_2_. Thus, it seems that the hydrophobicity of Mn_2_WO*_x_*/TiO_2_–SiO_2_ is decreased, which shall be not favourable for the water resistance and the activity. While the above activity experiments have demonstrated that the water resistance of the catalyst is enhanced, to explain the phenomenon, the water adsorption ability of the catalysts are measured under different temperatures. The results are listed in table 5. From the results, the water adsorption capacity of the Mn_2_WO*_x_*/TiO_2_–SiO_2_ samples are lower than the Mn_2_WO*_x_*/TiO_2_ at room temperature (RT) and decreased with the SiO_2_ content. Further, the difference is more pronounced at high temperature (100°C).

**Table 5.** The hydrophobicity of catalysts under different temperature.

samples	dry basis (g)	saturated with water at RT (g)	saturated with water at 100°C (g)	water adsorption capacity at RT (g g^−1^)	water adsorption capacity at 100°C (g g^−1^)
Mn_2_WO*_x_*/TiO_2_	0.5408	0.6081	0.5772	0.1244	0.0673
Mn_2_WO*_x_*/TiO_2_–SiO_2_(5)	0.5129	0.5755	0.5426	0.1221	0.0579
Mn_2_WO*_x_*/TiO_2_–SiO_2_(10)	0.4928	0.5450	0.5143	0.1059	0.0436
Mn_2_WO*_x_*/TiO_2_–SiO_2_(20)	0.6055	0.6650	0.6262	0.0983	0.0342

**Figure 10.**
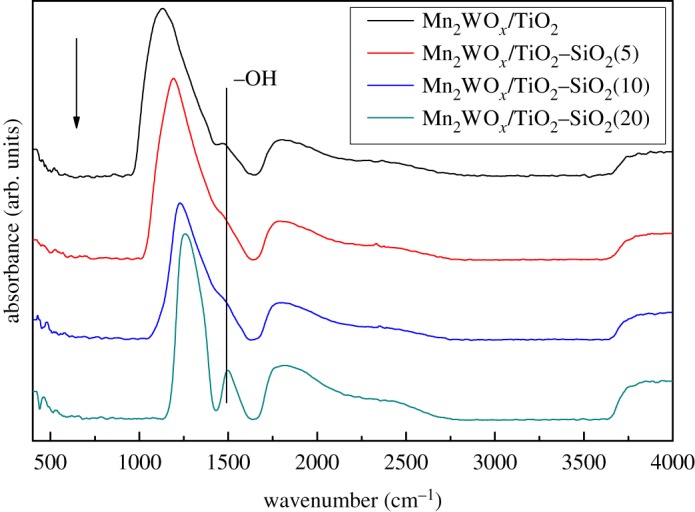
Skeleton FT-IR of the Mn_2_WO*_x_*/TiO_2_–SiO_2_ catalysts.

The weak water adsorption capacity of the Mn_2_WO*_x_*/TiO_2_–SiO_2_ catalysts may come from the formation of silicon hydroxyl. The skeleton FT-IR of the Mn_2_WO*_x_*/TiO_2_–SiO_2_ catalysts is then recorded and the results are shown in figure 10. It can be found that the hydroxyl vibration peaks at 1502 cm^−1^ are significantly changed after introduction of SiO_2_. For the Mn_2_WO*_x_*/TiO_2_–SiO_2_(20), it has changed from the shoulder peak to the independent peak, corresponding to its higher water adsorption. Meanwhile, the peak is weakened for the Mn_2_WO*_x_*/TiO_2_–SiO_2_(5) and the Mn_2_WO*_x_*/TiO_2_–SiO_2_(10) and their water adsorption content are somewhat lower too. Thus, the appropriate SiO_2_ content can reduce the amount of hydroxyl groups on the catalyst surface, and the excessive SiO_2_ increases the amount of hydroxyl groups on the catalyst surface.

### 3.4. Discussions

As mentioned earlier, this paper is intended to indirectly improve the SO_2_ resistance of the MnWO*_x_*/TiO_2_ catalyst by increasing its hydrophobicity. According to the activity tests, the SO_2_ and water resistance of the MnWO*_x_*/TiO_2_–SiO_2_ catalyst can be really improved under the premise that the NH_3_-SCR activity is maintained. The NO conversion is no longer decreased as soon as the catalyst contacts SO_2_, but it is maintained for some time on stream and then decreased slowly. Meanwhile, the water adsorption test shows that the water capacity of the catalyst decreases after SiO_2_ introduction.

**Figure 11.**
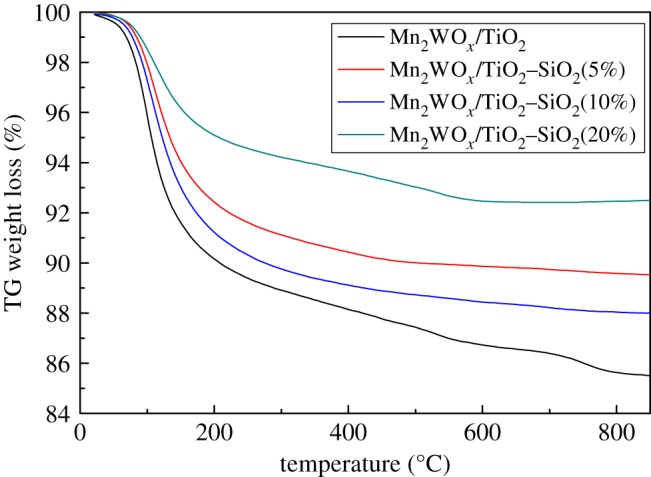
TG results of different Mn_2_WO*_x_*/TiO_2_–SiO_2_ catalysts after 600 min reaction.

However, it is a little hasty to make this assertion, as SiO_2_ introduction also changes some other properties of the catalyst. The specific surface area and pore volume of the MnWO*_x_*/TiO_2_–SiO_2_ catalysts is clearly larger than the MnWO*_x_*/TiO_2_. The decreasing degree of the specific surface area and pore volume of the MnWO*_x_*/TiO_2_–SiO_2_ catalysts after reaction is also lighter than the MnWO*_x_*/TiO_2_. Therefore, TG analysis has been performed after reaction and the results are displayed in figure 11. From the TG results, the water adsorption capacity of the Mn_2_WO*_x_*/TiO_2_–SiO_2_ catalysts is clearly lower than that of the Mn_2_WO*_x_*/TiO_2_. The weight loss of the Mn_2_WO*_x_*/TiO_2_–SiO_2_(5) and Mn_2_WO*_x_*/TiO_2_–SiO_2_(10) catalysts in higher temperature are also significantly lower than that of the Mn_2_WO*_x_*/TiO_2_. It may be helpful to prove that both the coke behaviour and hydrophobicity are restrained after SiO_2_ doping.

## 4. Conclusion

A series of TiO_2_–SiO_2_ mixed oxides supports were prepared by sol–gel method, and the MnWO*_x_* active components were loaded on the supports by liquid deposition method to form a series of MnWO*_x_*/TiO_2_–SiO_2_ catalysts. Owing to the reaction of manganese nitrate and ammonia tungstate to form MnWO_4_, the catalysts could be prepared at low temperature. The introduction of SiO_2_ in the TiO_2_ support increases the N_2_ selectivity of the MnWO*_x_*/TiO_2_ catalyst without decreasing the NH_3_-SCR activity. Meanwhile, with appropriate SiO_2_ (5% and 10% percentage of TiO_2_), the sulfur resistance of MnWO*_x_*/TiO_2_–SiO_2_ is clearly improved, the decreasing rate and degree of NO conversion are both slowed down and retarded. By a series of BET, XRD and TEM characterization, it can be inferred that the texture properties of MnWO*_x_*/TiO_2_–SiO_2_ are modified and the dispersion of MnWO*_x_* species is improved, which is beneficial for the NH_3_-SCR activity. Skeleton FT-IR, water adsorption capacity and XPS show that SiO_2_ introduction decreases the water adsorption capacity especially at the high temperatures, which might be favourable for the water and sulfur resistance of the catalyst.

## Data accessibility

Our data are available from Dryad Digital Repository: doi:10.5061/dryad.74pj317 [36].
